# Prevention challenges with current perceptions of HIV burden among HIV‐negative and never‐tested men who have sex with men in the Netherlands: a mixed‐methods study

**DOI:** 10.1002/jia2.25715

**Published:** 2021-08-27

**Authors:** Hanne ML Zimmermann, Ward PH van Bilsen, Anders Boyd, Maria Prins, Frenk van Harreveld, Udi Davidovich, HIV Transmission Elimination Team Amsterdam

**Affiliations:** ^1^ Department of Infectious Diseases Public Health Service of Amsterdam Amsterdam The Netherlands; ^2^ Stichting HIV Monitoring Amsterdam The Netherlands; ^3^ Department of Internal Medicine Division of Infectious Diseases Amsterdam Infection & Immunity Institute (AIII), Amsterdam UMC University of Amsterdam Amsterdam The Netherlands; ^4^ Department of Social Psychology University of Amsterdam Amsterdam The Netherlands; ^5^ National Institute for Public Health and the Environment (RIVM) Bilthoven The Netherlands

**Keywords:** HIV, sexual risk behaviour, men who have sex with men, prevention and control, burden

## Abstract

**Introduction:**

As biomedical advances improved HIV treatment, the perceptions of severity and anticipated consequences of HIV could have changed accordingly. This study investigates the current perceptions of severity and anticipated consequences of HIV infection and its association with sexual risk behaviour among HIV‐negative and never‐tested men who have sex with men (MSM) living in the Netherlands.

**Methods:**

In‐depth interviews with recently diagnosed HIV‐positive MSM were used to develop a questionnaire measuring the perceived severity and anticipated consequences of HIV infection. The questionnaire was distributed online between April and July 2019. A structural equation model was constructed to explore the anticipated consequences contributing to the perceived HIV severity and to assess the association between the perceived severity and sexual risk behaviour.

**Results:**

In total, 1,072 HIV‐negative and never‐tested MSM completed the questionnaire, of whom 28% reported recent sexual risk behaviour. Almost one‐quarter of participants (23%) had a low perceived HIV severity, which was associated with more prevalent sexual risk taking (β = −0.07, 95% CI = −0.12/−0.01). In this model, the perceived severity of HIV was more strongly associated with anticipated psychological consequences of HIV (β = 0.34, 95% CI = 0.24 to 0.44) and to a lesser extent with anticipated negative consequences of HIV on sex/relationships (β = 0.28, 95% CI = 0.19 to 0.38) and disclosure‐related consequences (β = 0.16, 95% CI = 0.07 to 0.26). Health‐related consequences of HIV were not significantly associated with the severity perceptions (β = 0.06, 95% CI = −0.03 to 0.14).

**Conclusions:**

Anticipated negative social and psychological consequences of HIV mostly contribute to high HIV‐severity perceptions in MSM. A smaller subgroup of MSM does not perceive HIV as a serious disease, which is associated with increased sexual risk taking. Efforts to normalize living with HIV are essential but might present a challenge for HIV prevention as it could, for a minority of MSM, decrease the motivation to prevent HIV infection.

## 1 INTRODUCTION

In most Western countries, men who have sex with men (MSM) are disproportionately affected by HIV [1]. For decades, condom use was the only prevention strategy promoted among MSM to prevent HIV acquisition. More recently, increased testing and treating of HIV‐positive individuals (i.e. “treatment as prevention”) and use of pre‐ and post‐exposure prophylaxis (PrEP and PEP respectively) in HIV‐negative individuals at‐risk of HIV have been implemented as additional prevention strategies. Theoretically, these strategies have the potential to eliminate new HIV infections among MSM [2]. Several socio‐cognitive and practical aspects surrounding HIV prevention uptake can make HIV elimination challenge. Socio‐cognitive aspects affecting health‐related behaviours, such as HIV prevention uptake, have been conceptualized in the Health Belief Model and Common‐Sense Model of Self‐Regulation, which suggests that health‐related behaviours are driven by, among others, their perceived benefits and costs/barriers, as well as perceptions on disease susceptibility and severity [3, 4].

In the current biomedical era of highly tolerable and effective treatment, one could assume that the severity of HIV can be justifiably perceived as less threatening for one’s physical wellbeing, which in turn might contribute to changes in motivation to engage in HIV‐protective behaviours. The perceptions concerning the severity of an illness, however, also relate to their anticipated social consequences [3, 5]. Numerous studies have reported the ongoing social consequences of HIV infection, such as stigma [6, 7, 8], and hence HIV cannot be viewed merely as a medical condition. Efforts have been made to reduce stigma and “normalize” living with HIV [9]. These efforts might lower the anticipated social consequences of HIV, which may give rise to lower HIV prevention uptake. In this study, we, therefore, revisit questions around the perceived HIV severity and investigate which perceptions of anticipated consequences of HIV contribute to the perceived severity of HIV among MSM living in the Netherlands. We, moreover, aimed to investigate the association between HIV‐severity perceptions and sexual risk taking.

## 2 METHODS

### 2.1 Study design and population

The perceptions on severity and anticipated consequences of living with HIV were investigated in a convenience sample of HIV‐negative and never‐tested MSM in an online survey. The development of the survey has been described previously [7]. Briefly, burdensome aspects of living with HIV were identified from in‐depth interviews with 18 Dutch MSM diagnosed with HIV between 2014 and 2018. The findings from these interviews were translated into quantifiable questionnaire items to measure the severity and anticipated consequences of HIV infection in HIV‐negative and never‐tested MSM. Members of the Dutch Association of People with HIV (Hiv Vereniging) and MSM community reviewed the questionnaire on acceptability and terminology. The questionnaire was subsequently piloted by a group of six HIV researchers at the Public Health Service of Amsterdam who were unrelated to the study. The survey was distributed online at gay dating sites/apps (Grindr, Planet Romeo) and via social media (Instagram, Facebook) between April and July 2019.

### 2.2 Study variables

The survey consisted of 38 items (Supplement S1). The first set of questions assessed socio‐demographic characteristics, such as gender, age, zip code, country of birth, relationship status, gender of sex partner(s), having HIV‐positive acquaintances and presence of any chronic disease other than HIV. We asked the result of the most recent HIV test (positive, negative or never tested). Questions were asked on sexual behaviour within the preceding year. Sexual risk behaviour was defined as having had condomless anal sex (CAS) with either a casual partner who was HIV positive with a detectable HIV viral load (VL) or a partner of unknown HIV status. Sexual behaviour was not considered risky with respect to HIV if current PrEP use was reported or if CAS was reported in a steady relationship, with a self‐reported HIV‐negative casual partner or with an HIV‐positive casual partner with an undetectable VL.

The perceptions of the general severity and anticipated consequences of HIV infection were assessed by 7‐point Likert items. Based on Chard *et al*. [10], the general perceived severity of HIV infection was assessed by the question *“How serious for you would it be if you had contracted HIV?”*, which could be answered from not bad at all (1) to very bad (7).

Items assessing the anticipated burden of HIV infection were categorized into the following themes based on in‐depth interviews: health, psychosocial, disclosure‐related and sex and relationship consequences. Responses to items evaluating consequences of HIV ranged from anticipating no/low burden (1) to anticipating high burden (7). Cronbach’s alpha (α) was used to examine the internal consistency between responses to questions within the same theme and items were combined if α was ≥0.8 [7].

*Health‐ and ART‐related items* consisted of expecting side‐effects from ART, difficulty integrating ART in one’s daily routine, difficulty remembering to take ART, difficulty of taking ART in the presence of others, habituating to ART taking and burden of hospital visits. The perceptions of getting sick more easily and having a shortened life‐expectancy if HIV positive was also categorized as health related.

*Psychosocial‐related items* consisted of expecting acceptance of being HIV positive, pre‐occupation with HIV, the impact of HIV on quality of life, change in relationship with family/friends, fear of infecting family/friends, being discriminated against, getting fired from employment, problems during travel, within the healthcare system or with obtaining a mortgage and onset or worsening of the following emotions if HIV positive: feelings of inferiority, loneliness, insecurity about the future, fear, sadness, feeling less attractive, shame, stress and anger.

*Disclosure‐related items* included expecting difficulty with disclosure and not disclosing (i.e. keeping HIV‐positive status a secret).

*Sex‐ and relationship‐related items* were asked with respect to steady and casual partners. The anticipated impact of HIV‐positive status on steady relationships was evaluated among all participants by questions on the expected change in the quality of relationships post diagnosis, difficulty of engaging in a serious relationship with someone, and being left by a steady partner. For participants in a steady relationship, the anticipated impact of HIV on sex with steady partners was assessed by asking to what extent one’s sex life would be expected to be worse and whether there would be less enjoyment of sex and more fear/stress during sex. The anticipated impact of HIV on sex with casual partners consisted of getting rejected by potential sex partners, difficulty finding new casual partners, reduced quality and enjoyment of sex with casual partners, and more fear/stress during sex with casual partners. The anticipated fear of infecting steady or casual sex partners was additionally categorized as sex related.

### 2.3 Statistical analyses

All analyses included MSM who reported being HIV negative at their last HIV test or MSM who had never been tested for HIV. Due to highly skewed distributions, responses to all perception items were re‐categorized as follows: 1 to 2, defined as no/low burden; 3 to 5, defined as neutral/medium burden; and 6 to 7, defined as high burden. This categorization was used in all analyses unless stated otherwise. We constructed a structural equation model (SEM) [11, 12] to (1) explore which anticipated consequences contributed to the general perceived severity of HIV infection and (2) assess the association between perceived severity and sexual risk behaviour. We chose this analytical approach because it allowed us to simultaneously model and test the interrelatedness of these two components.

1. Association between anticipated consequences and perceived severity of HIV
  We assumed that each of the four themes could be expressed as latent variables measured by responses to questions within themes. In order to determine which measurement variables to include, we regressed general perceived severity of HIV [dichotomized as perceiving HIV as very bad (score 6 to 7) or not bad or neutral (score 1 to 5)] on responses to perceived consequences of HIV per theme using logistic regression. All variables associated with a *p* < 0.2 in the univariable model were included in a multivariable model, from which all non‐significant variables were removed in a backward‐stepwise fashion. Variables from these models were used as measurement variables of the latent themes, whereas the latent variables were regressed as direct paths to the perceived severity of HIV in the SEM.
2. Association between perceived severity of HIV and sexual risk behaviour We then generated a path between the general perceived severity of HIV and sexual risk behaviour. To determine the exogenous variables that could influence the parameter estimate of this path, we regressed (i) any sexual risk behaviour on several socio‐demographic variables and (ii) general perceived severity of HIV (dichotomized) on socio‐demographic variables, both using logistic regression. We included variables associated with a *p* < 0.2 in univariable analysis and removed all non‐significant variables in a backward‐stepwise fashion. Socio‐demographic variables from these models were used as exogenous variables on paths to sexual risk behaviour and/or perceived severity of HIV in the SEM. Since the latent variables could induce confounding between general perceived severity and sexual risk behaviour, we included paths from each of latent variables, separately in four additional SEMs, to sexual risk behaviour and from socio‐demographic exogenous variables in *post hoc* analysis.

​

Parameter estimates (β) were standardized and estimated alongside their 95% confidence intervals (CI) using maximum likelihood methods. We tested if βs were greater than null using a Wald χ^2^ test.

Sensitivity analyses were performed in which CAS with an HIV‐negative casual partner without the use of PrEP was considered as sexual risk behaviour since individuals with an undiagnosed HIV infection might disclose being HIV negative.

All analyses were performed in Stata IC v15.0. A *p* < 0.05 was considered statistically significant.

### 2.4 Ethical considerations

The study was reviewed and approved by the Amsterdam University Medical Center ethics board. The participation in the survey was voluntary and anonymous. All participants consented to study participation.

## 3 RESULTS

In total, 1,072 HIV‐negative MSM completed the online survey. Of them, 950 (89%) were HIV‐negative MSM and 122 (11%) never‐tested MSM (Table 1). The median age of participants was 42 years (interquartile range 29 to 54 years). The majority was born in the Netherlands (89%, n = 958), had a college or university degree (61%, n = 658) and did not have a chronic illness (85%, n = 906). Approximately half reported having HIV‐positive acquaintances (51%, n = 544) and being in a steady relationship (46%, n = 495). Current or past PrEP use was reported by 221 (21%) participants. In total, 178 (17%) participants engaged in sexual risk behaviour in the preceding year. When CAS with an HIV‐negative partner without the use of PrEP was also considered as risk behaviour, the proportion of individuals with sexual risk behaviour increased to 28% (n = 304). Supplement S2 provides details on PrEP use and sexual behaviour among participants who reported sexual risk behaviour.

**Table 1:** Socio‐demographics and sexual risk behaviour of 1,072 HIV‐negative and never‐tested MSM living in the Netherlands

	N	%
Sociodemographics		
Age, median [IQR]	42	[29 to 54]
Born in the Netherlands	958	89%
Residences in one a large city^a^	359	33%
College degree or higher	658	61%
Having steady partner	495	46%
Having chronic disease other than HIV	166	15%
Having HIV‐positive acquaintance	544	51%
HIV test behaviour		
Never tested for HIV	122	11%
Sexual risk behaviour		
Sexual risk behaviour^b^ in preceding 6 months	299	28%
PrEP use		
Never used	851	79%
Past use	29	3%
Current use	192	18%
Condomless anal sex (CAS) in the preceding six months		
No	310	29%
Only with steady partners	276	26%
Only with casual partners	298	28%
With both steady and casual partners	188	18%

^a^ Large city includes Amsterdam, Rotterdam, Den Haag and Utrecht

^b^ sexual risk behaviour was defined as having had CAS with either a casual partner who was HIV positive with a detectable HIV viral load (VL) or a partner of unknown HIV status. Sexual behaviour was not considered risky with respect to HIV if current PrEP use was reported or if CAS was reported in a steady relationship, with a self‐reported HIV‐negative casual partner, or with an HIV‐positive casual partner with an undetectable VL.

### 3.1 Perceptions on severity and anticipated burden of HIV

The majority of MSM reported a high perceived severity of HIV (77%, n = 826; Table 2). The mean score (on a 7‐point Likert scale) of perceived severity was 6.1 (*SD* = 1.35). PrEP users reported lower perceived severity of HIV compared to non‐PrEP users (66% vs. 80%, *p* < 0.001).

**Table 2:** Perceived seriousness and anticipated consequences of living with HIV among 1,072 HIV‐negative and never‐tested MSM living in the Netherlands

	Low burden^a^ (n; %)	Neutral or medium burden^a^ (n; %)	High burden^a^ (n; %)
Perceived severity of HIV			
Perceived severity of HIV	Not bad (43; 4%)	Neutral (203; 19%)	Very bad (826; 77%)
Health‐ and ART‐related consequences			
Burdensome side effects	No (159; 15%)	Maybe (630; 59%)	Yes (283; 26%)
Integrating ART in daily routine	Easy (425; 40%)	Nor easy nor difficult (483; 45%)	Difficult (164; 15%)
Remembering daily ART taking	Easy (399; 37%)	Nor easy nor difficult (500; 47%)	Difficult (173; 16%)
Taking ART in presence of others	Easy (202; 19%)	Nor easy nor difficult (439; 41%)	Difficult (431; 40%)
Habituation of ART taking	Yes (522; 49%)	A little (394; 37%)	No (156; 15%)
Burden of hospital visits	No (257; 24%)	Neutral (531; 50%)	Yes (284; 26%)
Being more vulnerable for diseases due to HIV	No (141; 13%)	Neutral (512; 48%)	Yes (419; 39%)
Having shortened life expectancy due to HIV	No (268; 25%)	Neutral (520; 49%)	Yes (284; 26%)
Psychosocial consequences			
Acceptance of having HIV	Yes (332; 31%)	Neutral (449; 42%)	No (291; 27%)
Emotional burden^a^	Rarely (79; 7%)	Sometimes (437; 41%)	Often (556; 52%)
Pre‐occupation with HIV	Rarely (73; 7%)	Sometimes (496; 46%)	Often (503; 47%)
Quality of life	Improvement (31; 3%)	Unchanged (703; 66%)	Worsening (338; 32%)
Change in relationship with family/friends	Improvement (48; 4%)	Unchanged (848; 79%)	Worsening (176; 16%)
Fear to infect family/friends	No (572; 53%)	Neutral (256; 24%)	Yes (244; 23%)
Discrimination	Rarely (79; 7%)	Sometimes (554; 52%)	Often (439; 41%)
Getting fired	Rarely (399; 37%)	Sometimes (510; 48%)	Often (163; 15%)
Problems with healthcare system^b^	Rarely (259; 24%)	Sometimes (592; 55%)	Often (221; 21%)
Problems with obtaining mortgage	Rarely (208; 19%)	Sometimes (465; 43%)	Often (399; 37%)
Problems/limitations when traveling	Rarely (235; 22%)	Sometimes (594; 55%)	Often (243; 23%)
Disclosure‐related consequences			
Disclosure^c^	Low burden (61; 6%)	Medium burden (410; 38%)	High burden (601; 56%)
Non‐disclosure^c^	Low burden (65; 6%)	Medium burden (516; 48%)	High burden (491; 46%)
Disclose over time	Gets easier (256; 24%)	Remains unchanged (721; 67%)	Gets more difficult (95; 9%)
Sex‐ and relationship‐related consequences			
Difficulty getting steady relationship	No (93; 9%)	Neutral (411; 38%)	Yes (568; 53%)
Getting left by steady partner	Rarely (198; 18%)	Sometimes (559; 52%)	Often (315; 29%)
Quality of steady relationship	Improvement (38; 4%)	Unchanged (709; 66%)	Worsening (325; 30%)
Worsened sex life with steady partner^d^	No (111; 22%)	Neutral (224; 45%)	Yes (160; 32%)
Less enjoyment of sex with steady partner^d^	No (122; 25%)	Neutral (221; 45%)	Yes (152; 31%)
More fear/stress during sex with steady partner^d^	No (77; 16%)	Neutral (213; 43%)	Yes (205; 41%)
Getting rejected by potential casual partner	Rarely (42; 4%)	Sometimes (370; 35%)	Often (660; 62%)
Easier finding new casual partners	Yes (90; 8%)	Neutral (461; 43%)	No (521; 49%)
Worsened sex life with casual partners	No (174; 16%)	Neutral (493; 46%)	Yes (405; 38%)
Less enjoyment during sex with casual partners	No (208; 19%)	Neutral (511; 48%)	Yes (353; 33%)
More fear/stress during sex with casual partners	No (145; 14%)	Neutral (496; 46%)	Yes (431; 40%)
Fear to infect sex partners	No (249; 23%)	Neutral (348; 32%)	Yes (475; 44%)
Perceived insight			
Perceived insight in living with HIV	Little insight (227; 21%)	Neutral (553; 52%)	Lot of insight (292; 27%)

^a^ Emotions included feelings of inferiority, loneliness, insecurity about the future, fear, relief, depressive feelings, feeling less attractive, shame, stress and anger/frustration

^b^ problems with healthcare systems included problems with non‐HIV healthcare providers, health insurances, pharmacies and dentists

^c^ expected burden of (non‐)disclosure included burden of (non‐)disclosure to family, friends, fellow students, colleagues, existing steady partner, new steady partner, existing casual sex partner, and new casual sex partner

^d^ among those who reported to currently be in a steady relationship.

Regarding health‐related consequences, neutral or medium anticipated burden was most frequently reported. The anticipated consequence that was reported most burdensome was the perception of being more susceptible to other diseases due to HIV (39%, n = 419). A total of 284 (26%) participants believed that HIV would shorten their life expectancy. Difficulty with taking ART in presence of others was expected by 431 (40%) participants whereas integrating ART in one’s daily routine and remembering to take ART daily was less frequently expected to be burdensome (15%, n = 164; 16%, n = 173 respectively). In total, 26% of participants expected burden from ART‐related side‐effects (n = 283) or from HIV‐related hospital visits (n = 284).

The psychological consequences of HIV consisted mainly of expecting onset or worsening of negative emotions (52%, n = 556) and expecting preoccupation with HIV once becoming HIV positive (47%, n = 503). Almost one‐third of participants expected worsening quality of life once becoming HIV positive (32%, n = 338,) and did not expect being able to accept their HIV status (27%, n = 291). Most participants did not expect that being HIV positive would alter their relationship with family or friends (79%, n = 848) and did not fear infecting them (53%, n = 572). Frequent HIV‐related discrimination was expected by 439 (41%) participants, which was higher than expecting problems with obtaining a mortgage (37%, n = 399), during travelling (23%, n = 243) and with the healthcare system (21%, n = 221).

A high proportion of participants expected medium or high burden related to HIV disclosure if being HIV positive (38%, n = 410 and 56%, n = 601 respectively). Similarly, the medium or high burden of non‐disclosure was reported by 516 (48%) and 491 (46%) participants respectively.

Regarding sex‐ and relationship‐related consequences of HIV, the majority believed that they would often be rejected by potential casual sex partners once becoming HIV positive (62%, n = 660) and it would be more difficult to establish a steady relationship (53%, n = 568). Almost one‐third believed that a (potential) steady partner would leave them once becoming HIV positive (29%, n = 315) and that HIV would result in worsening of the quality of steady relationships (30%, n = 325). Fear of infecting sex partners was expected by 475 (44%) participants. A similar proportion expected fear or stress during sex with casual and sex partners (40%, n = 431 and 41%, n = 205 respectively).

### 3.2 Association between anticipated consequences and perceived severity of HIV

**Figure 1:**
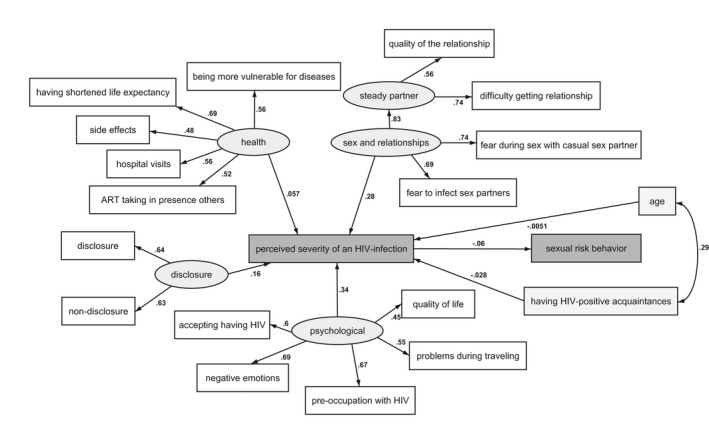
Structural equation model on associations between anticipated consequences of an HIV infection, perceived HIV severity and sexual risk behaviour among 1,072 HIV‐negative and never‐tested MSM living in the Netherlands. The numbers next to each pathway indicate the regression coefficient (β). The β regression coefficients generally represent the change in modelled outcomes (i.e. ending vertex of the path) per 1 unit increase in the independent variable (i.e. the starting vertex of the path). For example the β between the latent variables and expected severity represents the change in severity level per 1 standard deviation increased in the latent variable.

When constructing the SEM, we further structured the latent variable representing sex and relationship‐related themes to be measured by responses to two questions from this theme and another latent variable representing questions related to steady partners, as this led to a better fitting model. We also included a parameter to estimate covariance between age and having HIV‐positive acquaintances. The path diagram representing the final SEM is given in Figure 1. The fit of the final SEM was mostly adequate (Supplement S3).

Anticipated psychosocial consequences of HIV were most strongly related to the perceived severity of HIV (β = 0.34, 95% CI = 0.24 to 0.44*; p* < 0.001). This latent theme was represented by negative emotions (β = 0.69, 95% CI = 0.66 to 0.72), pre‐occupation with HIV (β = 0.67, 95% CI = 0.64 to 0.70), acceptance of HIV (β = 0.60, 95% CI = 0.55 to 0.65), problems during travel (β = 0.55, 95% CI = 0.50 to 0.60) and deterioration in quality of life (β = 0.45, 95% CI = 0.40 to 0.50) (*p* < 0.001 for all).

Anticipated negative consequences of HIV on sex and relationships were also strongly related to the general perceived severity of HIV (β = 0.28, 95% CI = 0.19 to 0.38; *p* < 0.001; Figure 1). This latent theme was represented by anticipated fear during sex with casual sex partners and fear of infecting sex partners (β = 0.74, 95% CI = 0.69 to 0.78 and β = 0.69, 95% CI = 0.64 to 0.74 respectively; *p* < 0.001 for both). In addition, this theme was represented by a latent variable based on items related to steady partners, which constituted anticipated difficulty in establishing a relationship and worsening in quality of a steady relationship (β = 0.74, 95% CI = 0.71 to 0.79 and β = 0.56, 95% CI = 0.51 to 0.61 respectively; *p* < 0.001 for both).

Disclosure‐related consequences of HIV were also significantly related to the perceived severity of HIV (β = 0.16, 95% CI = 0.07 to 0.26*; p* = 0.001). This latent theme was represented by the anticipated burden of both disclosure and non‐disclosure (β = 0.64, 95% CI = 0.60 to 0.69 and β = 0.63, 95% CI = 0.58 to 0.68; *p* < 0.001 for both).

Finally, anticipated health and ART‐related consequences of HIV were not associated with the perceived severity of HIV (β = 0.06, 95% CI = −0.03 to 0.14; *p* = 0.18). This latent theme was represented by anticipating a shorter life expectancy (β = 0.69, 95% CI = 0.63 to 0.74), burden of hospital visits (β = 0.56, 95% CI = 0.52 to 0.61), being more susceptible to other diseases (β = 0.56, 95% CI = 0.52 to 0.61), taking ART in the presence of others (β = 0.52, 95% CI = 0.46 to 0.57) and side‐effects (β = 0.48, 95% CI = 0.43 to 0.53) (*p* < 0.001 for all).

### 3.3 Association between the perceived severity of HIV and sexual risk behaviour

In the final SEM, a lower general perceived severity of HIV was correlated with sexual risk taking (β = −0.07, 95% CI = −0.12/−0.01; *p* = 0.02; Figure 1), while accounting for age and having HIV‐positive acquaintances as exogenous variables. Both age and having HIV‐positive acquaintances were not associated with the general perceived severity of HIV in this model (β = −0.01, 95% CI = −0.06 to 0.05, *p* = 0.86; β = −0.03, 95% CI = −0.09 to 0.03, *p* = 0.35 respectively).

In *post hoc* analysis, we observed that the association between lower general perceived severity of HIV and sexual risk taking was maintained when accounting for the possible confounding effect of consequences on health‐ (β = −0.08, 95% CI = −0.14,−0.03; *p* = 0.004), psychosocial‐ (β = −0.08, 95% CI = −0.15, −0.01; *p* = 0.02), disclosure‐ (β = −0.06, 95% CI = −0.11, −0.01; *p* = 0.03), and sex‐ and relationship‐ (β = −0.06, 95% CI = −0.13,0.00; *p* = 0.06) related consequences as separate latent variables.

Sensitivity analyses using the broader sexual risk definition yielded a similar association between the perceived HIV severity and sexual risk taking (data not shown).

## 4 DISCUSSION

In this study, we sought to explore current perceptions of severity and potential consequences of HIV infection, and its association with sexual risk taking among 1,072 HIV‐negative and never‐tested MSM living in the Netherlands. The majority of our study population perceived HIV as a severe illness, most of which related to the anticipated burden of interpersonal aspects, such as disclosure interactions and negative sexual experiences. Approximately one‐quarter of participants perceived HIV as a non‐severe to moderate illness, which was associated with more prevalent risk taking. Aspects of living with HIV that were the most frequently anticipated as non‐burdensome included health‐related aspects, such as ART taking and hospital visits, and interactions with non‐sexual contacts.

Our data on the perceived severity of HIV are in line with other recent studies reporting that the majority of HIV‐negative MSM perceive HIV as a serious illness [10, 13]. Balán *et al*. reported that 88% of HIV‐negative American MSM who engaged in CAS were moderately or highly concerned about contracting HIV [13]. Another study reported high perceived severity of HIV infection among HIV‐negative MSM from different countries, including Australia, Brazil, Canada, Thailand, South Africa, the United Kingdom and the United States [10]. We build on these previous findings by showing that severity perceptions are predominantly driven by anticipated negative social consequences, despite efforts to reduce these consequences among MSM living with HIV [9]. The fact that health‐related aspects did not contribute to severity perceptions suggests that living with HIV is perceived as biomedically manageable, but is not enough to normalize HIV socially. Since anticipated HIV‐related stigma has been previously mentioned as a barrier to HIV prevention uptake [14, 15, 16, 17], normalizing HIV remains a public health priority.

The association between the perceived severity of HIV and sexual risk taking is in line with socio‐cognitive theories, such as the Health Belief Model and Common‐Sense Model of Self‐Regulation [3, 4]. Interestingly, a few studies conducted before the widespread availability of PrEP or treatment‐as‐prevention did not find this association [10, 13, 18, 19, 20]. One study even reported a higher perceived severity of HIV among MSM who engaged in CAS compared to those who did not, which was explained by a lower self‐efficacy and lower perception of social norms related to safer sex among MSM engaging in CAS [18]. The fact that these MSM were concerned about contracting HIV but still engaged in CAS would argue for novel HIV prevention methods, such as PrEP. In our study, where the use of PrEP was reported by approximately one‐fifth of participants, it is possible that PrEP uptake predominantly occurred among the subgroup of MSM who were worried about acquiring HIV and who were at‐risk for HIV. Although this study lacked data on severity perceptions and sexual risk behaviour before PrEP initiation, we found that PrEP users perceived HIV as less severe compared to non‐PrEP users at the time of study participation. It might be possible that PrEP use has an effect on severity perceptions, as others also showed that HIV and sexual related anxiety are reduced by PrEP [21, 22]. This, however, warrants further investigation.

While there are many known factors that might instigate sexual risk‐taking behaviour, such as contextual (e.g. drug use) or those related to social norms [23, 24], the association between the low perceived severity of HIV and sexual risk behaviour in the era of biomedical treatment and prevention could suggest a niche of sexual risk behaviour among HIV‐negative and never‐tested MSM. As this subgroup of men do not expect HIV infection to have major implications on their lives, they also do not seem to apply conventional or biomedical prevention strategies. Ongoing biomedical advances and successes in normalizing life with HIV could further result in lower perceived severity of HIV infection, and consequently, the group of low severity perception/high‐risk behaviour MSM might expand over time. For HIV prevention efforts, it is therefore important to monitor whether normalization of HIV, which is indeed desirable, has a negative side‐effect on the motivation for engaging in HIV‐protective behaviours. Such efforts should avoid hindering the HIV normalization process. One way to do so could be to provide realistic testimonials on the experience of men living with HIV and embed them in positive community mobilization efforts (e.g. “let’s end HIV”) [25, 26, 27]. It is important to avoid fear‐based tactics, but also provide a realistic perception of HIV severity and specific burdens as currently experienced by individuals with HIV [7].

This study is subject to some limitations. First, our study population might not be fully representative of the overall HIV‐negative and never‐tested MSM population living in the Netherlands or other countries. Our results should be, therefore, generalized with caution to other settings. Second, the SEM approach used herein assumes that the sample has a multivariate, normal distribution, which might not be the case. Still, model fit was for the most part adequate. Third, sexual orientation and comprehensive measures of stigma and discrimination were not included in the questionnaire. We moreover only assessed the current use of PrEP rather than the use of PrEP during every CAS act and thus sexual risk behaviour could have been underestimated. Our risk definition is furthermore limited by the absence of data on negotiated safety within steady relationships and the validity of the perceived HIV status of casual partners. The effect of the latter is, however, likely limited since sensitivity analyses using different risk definitions yielded similar results.

## 5 CONCLUSIONS

The majority of HIV‐negative and never‐tested MSM perceive HIV as a severe illness, which mostly relates to anticipated negative social consequences of HIV. The minority of MSM who do not perceive HIV as a serious disease are more likely to engage in sexual risk behaviour. With ongoing biomedical advances and efforts to reduce HIV‐related stigma, this group might expand over time. Any intervention to increase the motivation to use HIV prevention strategies requires a balance between, on the one side, stigma reduction and normalization of HIV, whereas on the other, preserving the motivation to avoid HIV infection and adopt protective strategies. It could be one of the bigger challenges facing HIV prevention today.

## Competing interest

UD reports obtaining unrestricted research grants and speaker’s fees from Gilead Sciences, paid to his institute (Public Health Service of Amsterdam). MP received unrestricted research grants and speaker’s fees from Gilead Sciences, Roche, Abbvie, and MSD, paid to her institute (Public Health Service of Amsterdam). A.B. reports grants from ANRS and grants from Sidaction, outside the submitted work. All other authors declare no competing interests.

## Authors’ contributions

WvB, HZ and UD contributed to study concept and design. WvB and HZ performed all data analyses. AB, MP, FvH and UD supervised the data analysis and contributed to the interpretation of the data. WvB and HZ wrote the first draft of the report. All authors critically revised the manuscript and approved the final version for publication.

## Supporting information

**Supplement S1.** Questionnaire on the perceived severity and consequences of HIV and sexual risk behavior among MSM living in the Netherlands.
**Supplement S2**. PrEP use and sexual behavior among HIV‐negative and never‐tested MSM who engaged in sexual risk behavior during the preceding year.
**Supplement S3**. Fit statistics of the structural equation model on perceived severity and consequences of HIV on sexual risk behavior among 1,072 HIV‐negative and never‐tested MSM living in the Netherlands.
**Supplement S4**. Acknowledgements of the H‐TEAM consortium.
