# Supplementary material for: Prevention challenges with current perceptions of HIV burden among HIV‐negative and never‐tested men who have sex with men in the Netherlands: a mixed‐methods study
Source: J Int AIDS Soc. 2021 Aug 27;24(8):e25715. doi: 10.1002/jia2.25715 (PMC8395388; doi:10.1002/jia2.25715)
Supplement: Supplementary file 1 — Supplement S1. Questionnaire on the perceived severity and consequences of HIV and sexual risk behavior among MSM living in the Netherlands. Supplement S2. PrEP use and sexual behavior among HIV‐negative and never‐tested MSM who engaged in sexual risk behavior during the preceding year. Supplement S3. Fit statistics of the structural equation model on perceived severity and consequences of HIV on sexual risk behavior among 1,072 HIV‐negative and never‐tested MSM living in the Netherlands. Supplement S4. Acknowledgements of the H‐TEAM consortium. [file JIA2-24-e25715-s001.docx]

## Supplement 1. Questionnaire on the perceived severity and consequences of HIV and sexual risk behavior among MSM living in the Netherlands.

1. **What is your sex?** Male
    Female
    Other, namely _____________________________
2. **What was your sex at birth?**
    Male
    Female
3. **Who do you have sex with?**

Only with men

Only with women

Both with men and women

1. **In which year are you born?**

_______________________________

1. **Where are you born?**
    The Netherlands

West-Europe, other than the Netherlands

East-Europe

Surinam/Antilles

North-America

Central-America, other than the Antilles
 South-America, other than Surinam

Africa

Middle-East

Asia

Oceania

Other, namely __________________

1. **What are the first two numbers of your zipcode?** (If you do not want to answer this question, answer 99).
   _______________________________
2. **What is your highest completed education?** (If you completed an education abroad, choose an answer that comes closest to this education)
    No education

Primary education

Secondary education
 Secondary vocational education

Higher professional education
 University

Other, namely __________________

1. **Do you have a steady partner?** No

Yes

***The following questions will address the impact of HIV. Depending on your HIV status, questions will be asked in a way that suites your situation.***

1. **What was the test result of you latest HIV test?**

HIV positive

HIV negative

I have never been tested

1. **Do you have a chronic illness other than HIV?** No

Yes

1. **Do you know people in your direct environment who are living with HIV?** No

Yes

**[For HIV-negative MSM and MSM who never tested before only:]**

***The following questions will explore the impact you expect from living with HIV. Imagine how you would feel if you would have HIV.***

1. **The following questions explore the anticipated impact of taking HIV medication.**
   **How do you think it is to…
   … integrate pill taking in your daily routine?**Very easy 1 2 3 4 5 6 7 very difficult

**… remember/don’t forget HIV medication?**
Very easy 1 2 3 4 5 6 7 very difficult

**… take the HIV medication in presence of others?**
Not burdensome 1 2 3 4 5 6 7 very burdensome

1. **To what extent do you think you get used to daily pill taking?**Completely 1 2 3 4 5 6 7 not at all
2. **To what extent do you expect to experience side effects from the HIV medication?
   I expect…**no burdensome side effects 1 2 3 4 5 6 7 a lot of burdensome side effects
3. **To what extent do you agree with the following quotes?**

**If I would have HIV, I believe I …**

**… will get sick more easily / will be more vulnerable for other diseases.**Completely disagree 1 2 3 4 5 6 7 completely agree

**… would have a shorter life expectancy.**

Completely disagree 1 2 3 4 5 6 7 completely agree
**… will have more insight in/awareness of my health.**
Completely disagree 1 2 3 4 5 6 7 completely agree
**… will find the hospital visits burdensome.**

Completely disagree 1 2 3 4 5 6 7 completely agree
**… will find the hospital visits comforting.**

Completely disagree 1 2 3 4 5 6 7 completely agree

1. **To what extent do you think you get used to the HIV-related hospital visits?**

Completely 1 2 3 4 5 6 7 not at all

1. **To what extent do you think your quality of life will change due to HIV?**

**I expect that my quality of life will ...**improve 1 2 3 4 5 6 7 get worse

1. **To what extent do you think HIV will result in one of the following situations?**

**Discrimination / being treated adversely**
Never 1 2 3 4 5 6 7 often
**Getting rejected by a steady partner**Never 1 2 3 4 5 6 7 often
**Getting rejected by a potential new (sex-)partner**Never 1 2 3 4 5 6 7 often
**Getting fired / not getting hired by an employer**Never 1 2 3 4 5 6 7 often

1. **How difficult do you think it will be, in general, to tell that you are HIV positive to your:***(in case you don’t have some of the following contacts, imagine how it would be if you had these contacts)* **Family**not burdensome 1 2 3 4 5 6 7 very burdensome
   **Friends**
   not burdensome 1 2 3 4 5 6 7 very burdensome

**Colleagues / work-related people**
not burdensome 1 2 3 4 5 6 7 very burdensome

**Fellow students**
not burdensome 1 2 3 4 5 6 7 very burdensome

**New steady partner(s)**
not burdensome 1 2 3 4 5 6 7 very burdensome

**Existing steady partner(s)**

not burdensome 1 2 3 4 5 6 7 very burdensome **New sex partner(s)**
not burdensome 1 2 3 4 5 6 7 very burdensome

**Existing steady partner(s)**not burdensome 1 2 3 4 5 6 7 very burdensome

1. **To what extent do you think the burden of telling others that you are HIV positive will change over time?
   I expect that, over time, it will be …**

Easier 1 2 3 4 5 6 7 more difficult

1. **We can imagine that you would not share your HIV status with everybody in case you would have HIV.
   How burdensome do you think it would be, in general, to keep your HIV status for yourself with your:**

*(in case you don’t have some of the following contacts, imagine how it would be if you had these contacts)*

**Family**not burdensome 1 2 3 4 5 6 7 very burdensome
**Friends**
not burdensome 1 2 3 4 5 6 7 very burdensome

**Colleagues / work-related people**
not burdensome 1 2 3 4 5 6 7 very burdensome

**Fellow students**
not burdensome 1 2 3 4 5 6 7 very burdensome

**New steady partner(s)**
not burdensome 1 2 3 4 5 6 7 very burdensome

**[For those with a steady partner:] Existing steady partner(s)**

not burdensome 1 2 3 4 5 6 7 very burdensome **New sex partner(s)**
not burdensome 1 2 3 4 5 6 7 very burdensome

**Existing steady partner(s)**not burdensome 1 2 3 4 5 6 7 very burdensome

1. **To what extent do you expect HIV will change the bond you have with your family or friends?** (In case you expect nothing will change, answer 4).

   I expect a better bond 1 2 3 4 5 6 7 I expect the bond will get worse
2. **To what extent do you think HIV will result in (an increase of) one of the following feelings/thoughts?**

**Feelings of inferiority**
Rarely 1 2 3 4 5 6 7 often

**Loneliness**

Rarely 1 2 3 4 5 6 7 often **Insecurity about the future**Rarely 1 2 3 4 5 6 7 often

**Living more conscious**

Rarely 1 2 3 4 5 6 7 often
**Fear**Rarely 1 2 3 4 5 6 7 often

**Relief**

Rarely 1 2 3 4 5 6 7 often **Depressive feelings**

Rarely 1 2 3 4 5 6 7 often

**Increased confidence**
Rarely 1 2 3 4 5 6 7 often

**Feeling less attractive**

Rarely 1 2 3 4 5 6 7 often **Shame**

Rarely 1 2 3 4 5 6 7 often **Stress**
Rarely 1 2 3 4 5 6 7 often
**Anger/frustration**
Rarely 1 2 3 4 5 6 7 often

1. **To what extent do you think HIV will take over your thoughts?**

Rarely 1 2 3 4 5 6 7 often

1. **Indicate to what extent you agree with the following quote.**

**If I would have HIV it would be harder to engage in a serious relationship with someone.**

Totally disagree 1 2 3 4 5 6 7 totally agree

1. **[For those with a steady partner:]
   To what extent do you think HIV will influenced your sexual life with your steady partner? Indicate to what extent you agree with the following quotes.

   When it comes to serious relationships, I expect HIV will result in:**

**a worse sex life**

Totally not agree 1 2 3 4 5 6 7 fully agree

**less enjoying sex**

Totally not agree 1 2 3 4 5 6 7 fully agree

**more fear/stress towards sex**

Totally not agree 1 2 3 4 5 6 7 fully agree

**more freedom in condom use**

Totally not agree 1 2 3 4 5 6 7 fully agree

**a more adventurous sex life**

Totally not agree 1 2 3 4 5 6 7 fully agree

1. **To what extent do you think HIV will affect the quality of your relationship(s)?**
   I expect the quality will …
   improve 1 2 3 4 5 6 7 get worse
2. **To what extent do you think HIV will influence your sexual life with your casual partner(s)? Indicate to what extent you agree with the following quotes. In case you don’t have any casual sex partners, imagine how it would be if you would have casual sex partners.

   With my casual sex partners, I expect HIV will result in:

    a worse sex life:**

Totally not agree 1 2 3 4 5 6 7 fully agree

**less enjoying sex:**

Totally not agree 1 2 3 4 5 6 7 fully agree

**more fear/stress towards sex:**

Totally not agree 1 2 3 4 5 6 7 fully agree

**more freedom in condom use:**

Totally not agree 1 2 3 4 5 6 7 fully agree
 **easier finding a sex partner:**
 Totally not agree 1 2 3 4 5 6 7 fully agree

**a more adventurous sex life:**

Totally not agree 1 2 3 4 5 6 7 fully agree

1. **Scientific research showed that people with HIV and an undetectable viral load can not sexually transmit HIV to someone else. To what extent do you trust on this?**
   Totally 1 2 3 4 5 6 7 not at all
2. **To what extent would you be afraid to transmit HIV to your family/friends/colleagues?**Totally not 1 2 3 4 5 6 7 a lot
3. **[For those with a steady partner:]
   To what extent would you be afraid to transmit HIV to your steady partner(s)?**
   Totally not 1 2 3 4 5 6 7 a lot
4. **To what extent would you be afraid to transmit HIV to your casual sex partner(s)?** *(in case you don’t have any casual sex partners, imagine how it would be if you had a casual sex partner)*
   Totally not 1 2 3 4 5 6 7 a lot
5. **To what extent do you think you will experience problems/limitations due to having HIV with the following?**

**Pharmacy**

Never 1 2 3 4 5 6 7 often

**Mortgage lender**

Never 1 2 3 4 5 6 7 often

**Traveling**

Never 1 2 3 4 5 6 7 often

**Health insurance**

Never 1 2 3 4 5 6 7 often
 **Dentist** Never 1 2 3 4 5 6 7 often

**Health care provider other than HIV-related health care provider**

Never 1 2 3 4 5 6 7 often

1. **How serious for you would it be if you had contracted HIV?**
   Not serious 1 2 3 4 5 6 7 very serious
2. **To what extent do you think you will accept being HIV positive?**

**I expect that I will …**fully accept it 1 2 3 4 5 6 7 not accept it at all

1. **To what extent do you think you have insight in what it is like to live with HIV?**No/little insight 1 2 3 4 5 6 7 a lot of insight
2. **Do you use PrEP*?** no, never used it
    no, but I have used it in the past
    yes

   *PrEP refers to pills that can be taken before sex to prevent becoming HIV infected. PrEP differs from PEP, which are pills that are taken after possible sexual exposure to HIV.
3. **Did you have anal sex without a condom with one or more sex partners in the preceding year?** no

yes, with my steady partner
 yes, with my casual partner (one-night stands, fuck buddies)
 yes, both with my steady partner as with casual partners

**In case you indicated to have had sex with casual partners: With what types of partners did you have condomless anal sex in the preceding year (multiple answers possible):**

partner(s) of whom I don’t know the HIV status
  HIV positive partner(s)

HIV negative partner(s)

**In case you indicated to have had sex with HIV-positive casual partners: What was the viral load of these partners?** Undetectable

Detectable

I don’t know

## Supplement 2. PrEP use and sexual behavior among HIV-negative and never-tested MSM who engaged in sexual risk behavior during the preceding year.

|  | Participants engaging in sexual risk behavior (*definition A*) (N=178) | | Participants engaging in sexual risk behavior (*definition B*)  (N=304) | |
| --- | --- | --- | --- | --- |
|  | n | % | n | % |
| **PrEP use** |  |  |  |  |
| never used | 169 | 95% | 288 | 95% |
| ever use | 9 | 5% | 16 | 5% |
| current use | 0 | 0% | 0 | 0% |
| **Condomless anal sex (CAS)** |  |  |  |  |
| no CAS | 0 | 0% | 0 | 0% |
| CAS with steady partner | 0 | 0% | 0 | 0% |
| CAS with casual partner(s) | 114 | 64% | 203 | 67% |
| CAS with steady and casual partners | 64 | 36% | 101 | 33% |
| **CAS with casual partners according to HIV-status** |  |  |  |  |
| CAS with HIV-negative casual partner | 66 | 37% | 192 | 63% |
| CAS with casual partner with unknown HIV-status | 178 | 100% | 178 | 59% |
| CAS with HIV-positive casual partner | 40 | 23% | 51 | 17% |
| undetectable viral load | 37 | 21% | 48 | 16% |
| detectable viral load | 9 | 5% | 9 | 3% |
| unknown viral load | 16 | 9% | 16 | 5% |

**Definition A**: Having had condomless anal sex with a casual partner who was HIV-positive with a detectable HIV viral load (VL) or a partner of unknown HIV status. Sexual behavior was not considered risky with respect to HIV if current PrEP use was reported or if condomless sex was reported in a steady relationship, with an HIV-negative casual partner, or with an HIV-positive casual partner with an undetectable VL.

**Definition B**: Having had condomless anal sex with a casual partner who was HIV-negative, HIV-positive with a detectable HIV viral load (VL), or a partner of unknown HIV status. Sexual behavior was not considered risky with respect to HIV if current PrEP use was reported, or if condomless sex was reported in a steady relationship or with an HIV-positive casual partner with an undetectable VL.

##

## Supplement 3. Fit statistics of the structural equation model on perceived severity and consequences of HIV on sexual risk behavior among 1,072 HIV-negative and never-tested MSM living in the Netherlands.

We used a likelihood ratio test to determine whether the final fit of the SEM was as good as a saturated model, defined as a model whose covariances are fitted perfectly. In addition, we examined the overall fit of the SEM by estimating the root mean squared error of approximation (RMSEA), for which a lower bound of the 90%CI above 0.05 and upper 90%CI bound below 0.10 indicate adequate fit. We also estimated the standardized root mean squared residual (SRMR) and the coefficient of determination (CD), for which values close to 0 and 1, respectively, indicate adequate fit.

There was evidence that the final SEM fit as well as a saturated model (*p*<0.001). The lower and upper bounds of the RMSEA were 0.118 and 0.125, respectively, and SRMR was 0.233; all suggesting slightly excessive variation in the final model. CD was 0.995, suggesting adequate fit with respect to variance explained by the model.

| ***Fit statistics*** | Differences with respect to modeling latent variable on sex and relationship | |
| --- | --- | --- |
|  | Model 1: no additional latent variables | Model 2: items with steady partners used as a latent variable |
| -log likelihood* | 21,827.8 | 21,812.7 |
| Bayesian information criteria | 44,046.4 | 44,023.0 |
| 90% confidence interval of root-squared error of approximation | 0.118-0.126 | 0.118-0.125 |
| Standardized root mean squared residual | 0.233 | 0.233 |
| Coefficient of determination | 0.993 | 0.995 |

*Likelihood ratio test (4 d.f.) reveals that Model 2 has a significantly lower likelihood than Model 1 (*p*<0.001), thus Model 2 has better fit.

## Supplement 4. Acknowledgements of the H-TEAM consortium

H-TEAM Steering Committee: J.E.A.M. van Bergen6;4;5, G.J. de Bree1;2, P. Brokx8, F. Deug6, M. Heidenrijk1, M. Prins3;2, P. Reiss1;7 (chair), M. van der Valk2

H-TEAM Core Project Group: J.E.A.M. van Bergen6;4;5, G.J. de Bree1;2 (chair), P. Brokx8, U. Davidovich3, S.E. Geerlings2, E. Hoornenborg3, A. Oomen6, A. van Sighem7, W. Zuilhof6 H-TEAM

Project Management: N. Schat1

H-TEAM additional collaborators: R.C.A. Achterbergh3, M. van Agtmael24, J. Ananworanich22, D. Van de Beek17, G.E.L. van den Berk11, D. Bezemer7, A. van Bijnen6, W.L. Blok11, S. Bogers2, M. Bomers24, C.A.B. Boucher13, W. Brokking26, D. Burger20, K. Brinkman11, N. Brinkman32, M. de Bruin12, S. Bruisten3, L. Coyer3, R. van Crevel29, C.G. Daans3;34, L. Dellemann6, M. Dijkstra3, Y.T. van Duijnhoven3, A. van Eeden26, L. Elsenburg26, M.A.M. van den Elshout3, C. Ester7, E. Ersan3, P. E.V. Felipa3, P.H.J. Frissen11, T.B.H. Geijtenbeek18, M.H. Godfried2, J. van Gool3, A. Goorhuis2, M. Groot26, M.L. Groot Bruinderink3, C.A. Hankins1, A. Heijnen30;31, M.M.J Hillebregt7, A. Hogewoning3, M. Hommenga3, J.W. Hovius2, Y. Janssen32, K. de Jong3, V. Jongen3, N.A. Kootstra19, R.A. Koup21, F.P. Kroon16, T.J.W. van de Laar35;36, F. Lauw37, M. M. van Leeuwen3, K. Lettinga27, I. Linde3, D.S.E. Loomans3, J.T. van der Meer2, T. Mouhebati6, B.J. Mulder3, J. Mulder25, F.J. Nellen2, A. Nijsters6, H. Nobel2, P. Oostvogel3, E.L.M. Op de Coul5, E. Peters24, I.S. Peters3, T. van der Poll2, O. Ratmann28, C. Rokx14, M.S. van Rooijen3, M.F. Schim van der Loeff3;10, W.E.M. Schoute11, G.J. Sonder3, J. Veenstra27, A. Verbon14, F. Verdult8, J. de Vocht24, H.J. de Vries3;9;10, S. Vrouenraets27, M. van Vugt2, W.J. Wiersinga2, F.W. Wit2;7, L.R. Woittiez2, S. Zaheri7, P. Zantkuijl6, M.C. van Zelm23, A. Żakowicz33, H.M.L. Zimmermann3.

1 Department of Global Health, Amsterdam UMC – location AMC, and Amsterdam Institute for Global Health and Development, Amsterdam, the Netherlands

2 Department of Internal Medicine, Division of Infectious Diseases, Amsterdam UMC – location AMC, Amsterdam, the Netherlands

3 Department of Infectious Diseases, Public Health Service of Amsterdam, Amsterdam, the Netherlands

4 Department of General Practice, Amsterdam UMC – location AMC, University of Amsterdam, Amsterdam, the Netherlands

5 Epidemiology and Surveillance Unit, Center for Infectious Disease Control, National Institute of Public Health and the Environment, the Netherlands

6 STI AIDS Netherlands, Amsterdam, the Netherlands

7 Stichting HIV Monitoring, Amsterdam, the Netherlands

8 Dutch Association of PLHIV, Amsterdam, the Netherlands

9 Department of Dermatology, Amsterdam UMC – location AMC, University of Amsterdam, Amsterdam, the Netherlands

10 Center for Infection and Immunology, Amsterdam (CINIMA), Amsterdam UMC – location AMC, University of Amsterdam, Amsterdam, the Netherlands

11 Department of internal medicine, OLVG – location East, Amsterdam, the Netherlands

12 Aberdeen Health Psychology Group, Institute of Applied Health Sciences, University of Aberdeen, Aberdeen, United Kingdom

13 Department of viro-science, Erasmus Medical Center Rotterdam, Rotterdam, the Netherlands

14 Department of Internal Medicine and Infectious Diseases, Erasmus Medical Center, Rotterdam, the Netherlands

16 Department of Infectious Diseases, Leiden University Medical Center, Leiden, the Netherlands

17 Center of Infection and Immunity Amsterdam (CINIMA), Department of Neurology, Amsterdam UMC – location AMC, Amsterdam, the Netherlands

18 Laboratory of Experimental Immunology, Amsterdam UMC – location AMC Amsterdam, the Netherlands

19 Laboratory for Viral Immune Pathogenesis, Amsterdam UMC – location AMC Amsterdam, the Netherlands

20 Department of Pharmacy, Radboud University Nijmegen Medical Center, Nijmegen, the Netherlands

21 Immunology Laboratory, Vaccine Research Center, NIAID, National Institutes of Health

22 US Military HIV Research Program and the Henry M. Jackson Foundation for the Advancement of Military Medicine, Bethesda, United States

23 Department of Virology, Erasmus Medical Center, Rotterdam, the Netherlands

24 Department of Internal Medicine, Amsterdam UMC – location VUMC, Amsterdam, the Netherlands

25 Department of Internal Medicine, Slotervaart Hospital, Amsterdam, the Netherlands

26 DC Clinics, Amsterdam, the Netherlands

27 Department of Internal Medicine, OLVG – location West , Amsterdam, the Netherlands

28 School of Public Health, Faculty of Medicine, Imperial College London, London, United Kingdom

29 Department of Internal Medicine, Radboud University Nijmegen Medical Center, Nijmegen, the Netherlands

30 Sexology Center Amsterdam, Amsterdam, the Netherlands

31 GP practice Heijnen & de Meij, Amsterdam, the Netherlands

32 Elaa – First line Amsterdam Almere, Amsterdam, the Netherlands

33 AIDS Healthcare Foundation, Amsterdam, the Netherlands

34 Center of Expertise on Gender Dysphoria, Amsterdam UMC – location VUMC, Amsterdam, the Netherlands

35 Department of Medical Microbiology, OLVG, Amsterdam, the Netherlands

36 Department of Donor Medicine Research, Laboratory of Blood-borne Infections, Sanquin Research, Amsterdam, the Netherlands

37 Department of Internal Medicine, Medical Center Jan van Goyen, Amsterdam, the Netherlands
